# Supplementary material for: Novel xylan-degrading enzymes from polysaccharide utilizing loci of Prevotella copri DSM18205
Source: Glycobiology. 2021 Jun 15;31(10):1330–49. doi: 10.1093/glycob/cwab056 (PMC8631079; doi:10.1093/glycob/cwab056)

Supplementary Data

Novel xylan degrading enzymes from polysaccharide utilizing loci of *Prevotella copri* DSM18205

Javier A. Linares-Pastén[1*], Johan Sebastian Hero[2], José Horacio Pisa[2], Cristina Teixeira[1], Margareta Nyman[3], Patrick Adlercreutz[1], M. Alejandra Martinez[2,4], Eva Nordberg Karlsson[1**]

[1]*Biotechnology, Dept of Chemistry, Lund University, P.O.Box 124, 221 00 Lund, Sweden.*
[2]*Planta Piloto de Procesos Industriales Microbiológicos PROIMI-CONICET, Av. Belgrano y Pasaje Caseros, T4001 MVB, San Miguel de Tucumán, Argentina.*
[3] *Dept Food Technology, Engineering and Nutrition, Lund University, P.O. Box 124, SE-221 00 Lund, Sweden.*
[4]*Facultad de Ciencias Exactas y Tecnología, UNT. Av. Independencia 1800, San Miguel de Tucumán, 4000, Argentina.*

Corresponding authors:
[*]e-mail: *javier.linares_pasten@biotek.lu.se*
[**] e-mail: *eva.nordberg_karlsson@biotek.lu.se*

S1. Modularity of the Polysaccharide Utilization Loci (PULs) predicted by the Polysaccharide-Utilization Loci DataBase (PULB) of *Prevotella copri* DMS 18205. **HTCS**: Hybrid Two-Component Systems sensor-regulator. **unk**: hypothetical protein. **GH**: Glycoside Hydrolase Family. **Sus**: Starch-Utilization System. **ECF-σ**: Extracytoplasmic Function Sigma Factor. **PL**: Polysaccharide Lyase Family. **CE**: Carbohydrate Esterase Family. **GT**: Glycosyl Transferase Family. **CBM**: Carbohydrate-Binding Module Family. **Pept_SB**: Peptidases Serine Family. **Anti-σ**: Extracytoplasmic Function Anti-Sigma Factor. **MFS**: Major Facilitator Superfamily permease.

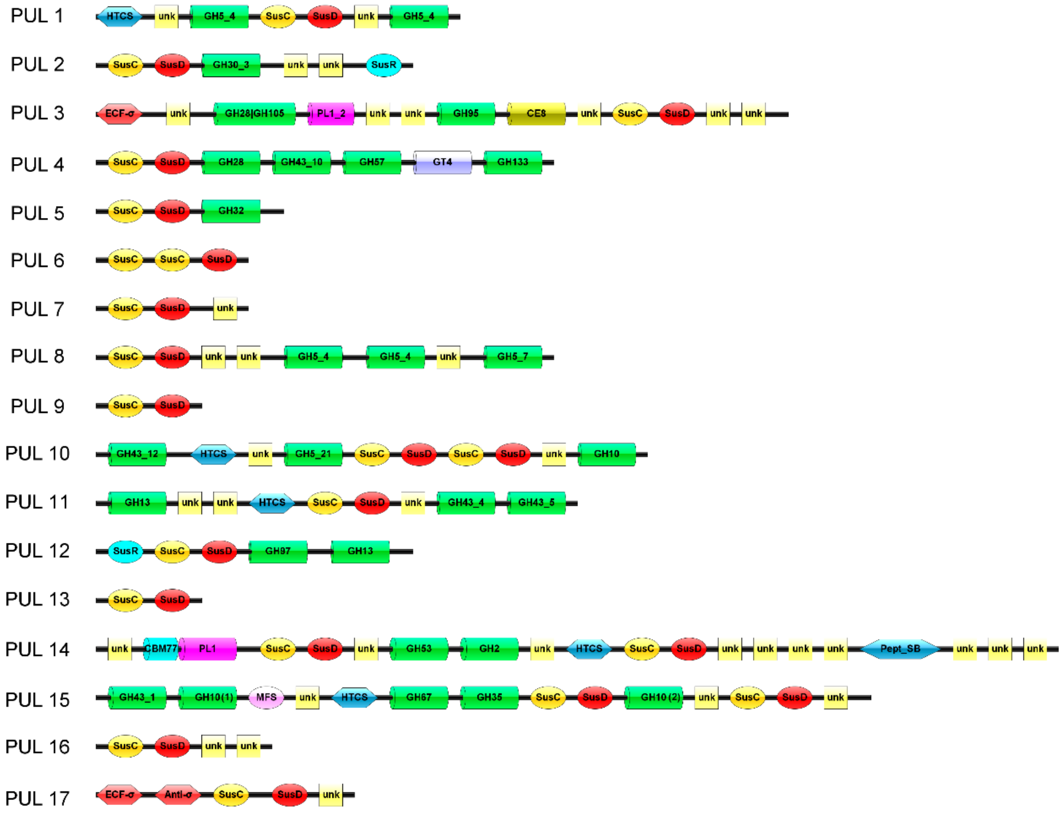

S2. SDS-PAGEs showing GH10 (1), GH43_1 and GH43_12 proteins from *Prevotella copri* DSM 18205 overexpressed and purified. Molecular weight marker in kDa (**M**), crude extract (**C**), and the purified protein from the soluble fraction (**P**).

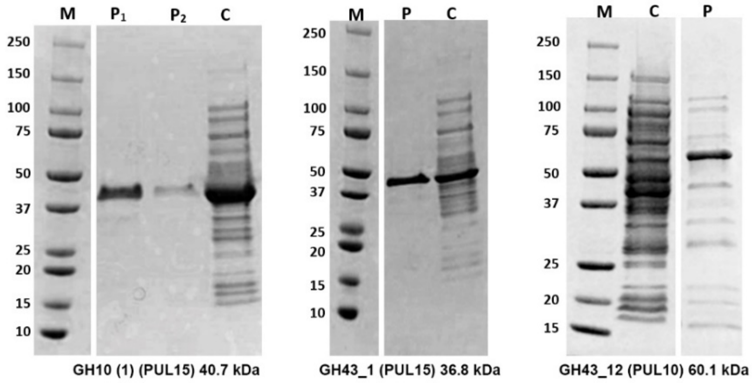

S3. Nonlinear regressions to the Michaelis–Menten equation at 37°C and pH 5.5. Error bars represent the standard deviation for n =3. (**A**) GH43_12 (PUL 10), substrate: p-NP-α-L-arabinofuranoside. (**B**) GH10 (1) (PUL 15), substrates: p-NP-Xyl2 (p-Nitrophenyl-β-xylobioside) and p-NP-Xyl3 (p-Nitrophenyl-β-xylotrioside). (**C**) GH43_1 (PUL 15), substrates: X2 (xylobiose); X3 (xylotriose); X4 (xylotetraose), X5 (xylopentaose) and X6 (xylohexaose).

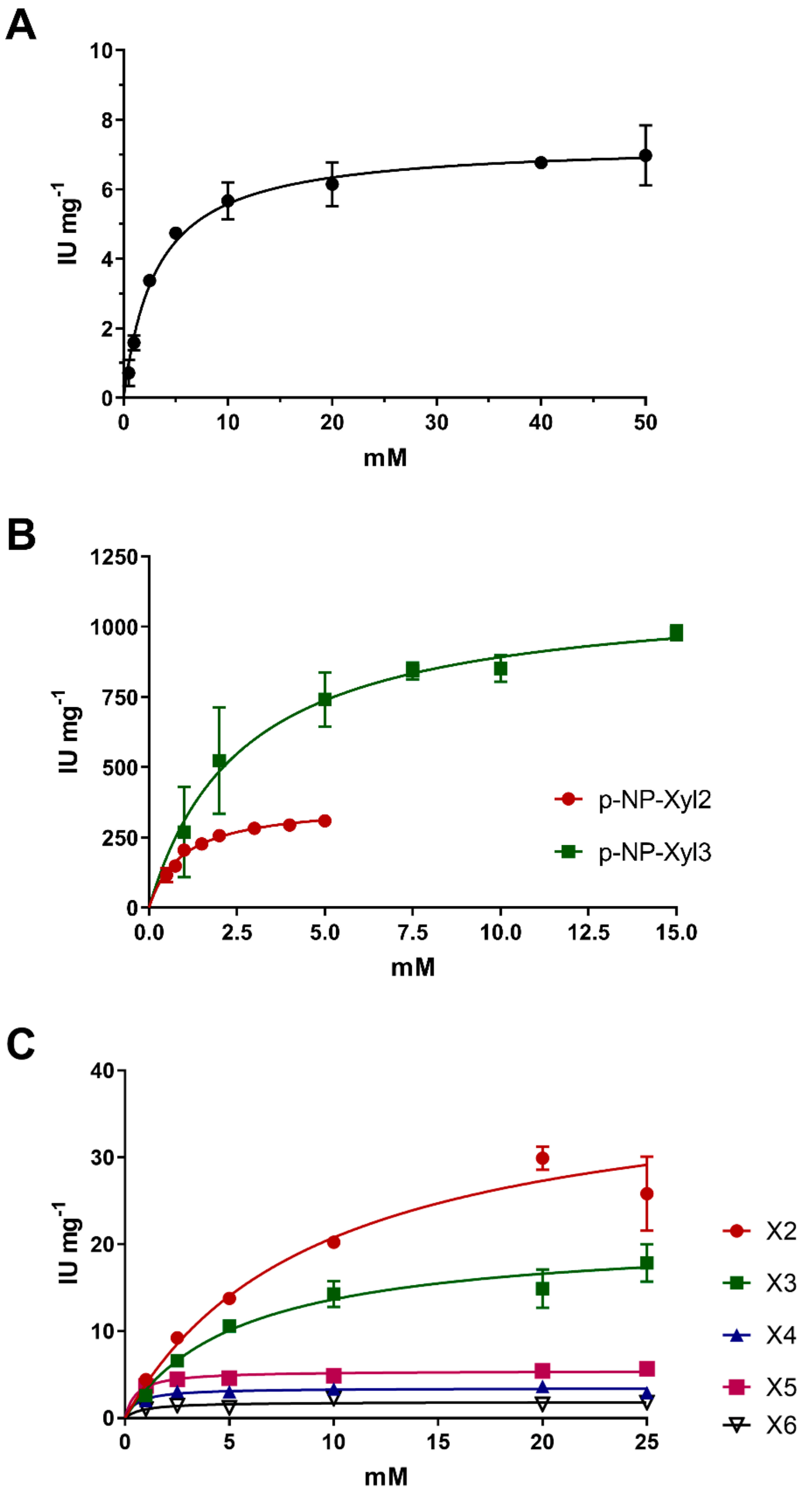

S4. Molecular models validation. Quality Z-scores of the hybrid models obtained using YASARA.

Structure	Dihedrals	Packing 1D	Packing 3D	Overall
GH43_12 (PUL 10)	-0.165	-0.680	-2.728	-1.558 satisfactory
GH43_1 (PUL 15)	0.891	-0.44	-2.617	-1.232 satisfactory
GH10 (1) (PUL 15)	0.120	0.565	-1.523	-0.470 good

S5. Chromatograms ofhydrolysis products from enzymatic degradation using the PUL15 enzymes from GH10 and GH43_1, along with substrate controls. Notice beechwood xylan consisted of oligomers which affected the yield of the enzymatic hydrolysis. Birchwood xylan (**A**), beechwood xylan (**B**), arabinoxylan (**C**) and quinoa xylan (**D**). X1, xylose; X2, xylobiose; X3, xylotriose; X4, xylotetarose; X5, xylopentaose.

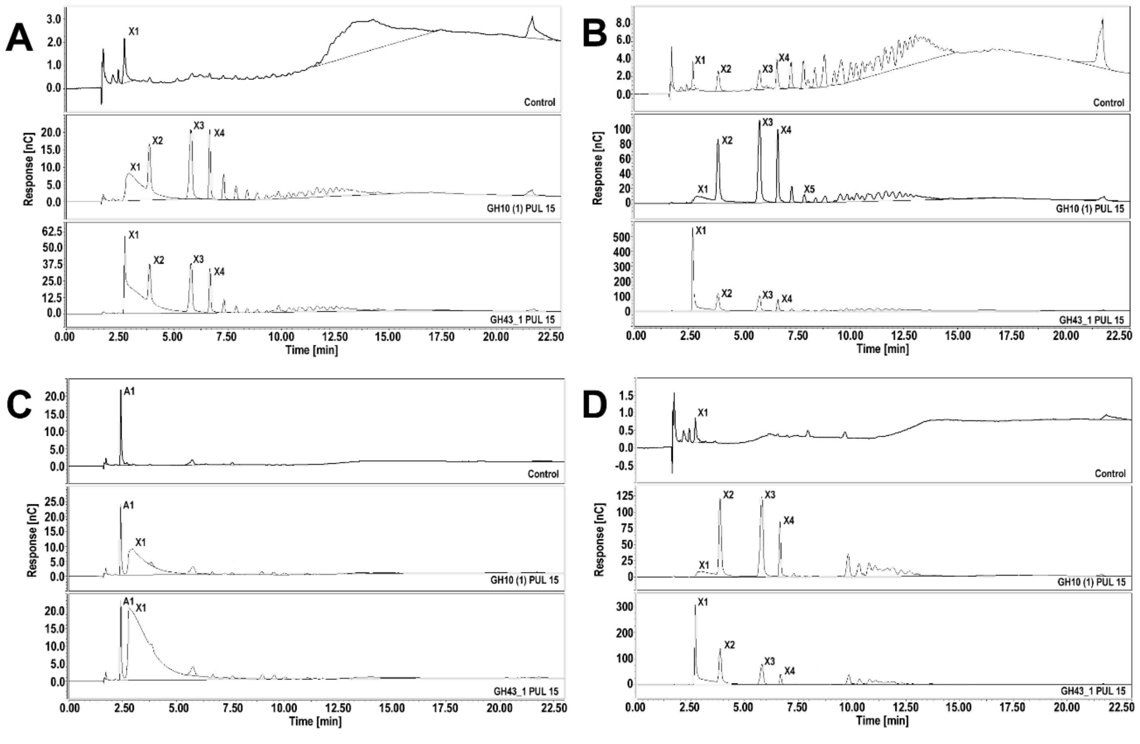

Supplement: SupplementaryData_cwab056 [file SupplementaryData_cwab056.pdf]
